# Supplementary material for: Evolution of fast root gravitropism in seed plants
Source: Nat Commun. 2019 Aug 2;10:3480. doi: 10.1038/s41467-019-11471-8 (PMC6677796; doi:10.1038/s41467-019-11471-8)
Supplement: Supplementary file 1 — Supplementary Information [file 41467_2019_11471_MOESM1_ESM.pdf]

Supplementary Information

Evolution of fast root gravitropism in seed plants

Zhang et al.

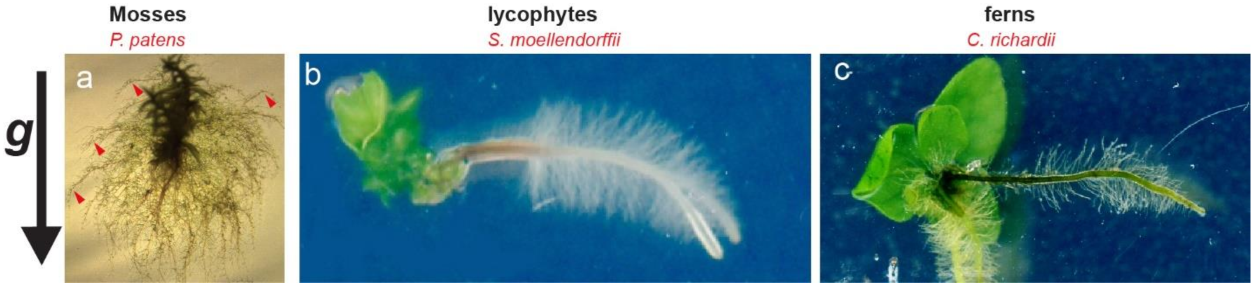

Supplementary Figure 1. Slow root gravitropism in the basal vascular plant lineages. a-c, The gravitropism analysis of the rhizoid of moss *P. patens* (**a**) and the roots of the basal vascular plants lycophyte *S. moellendorffii* (**b**) and the fern *C.richardii* (**c**) after a 90° plant reorientation for 5 days.

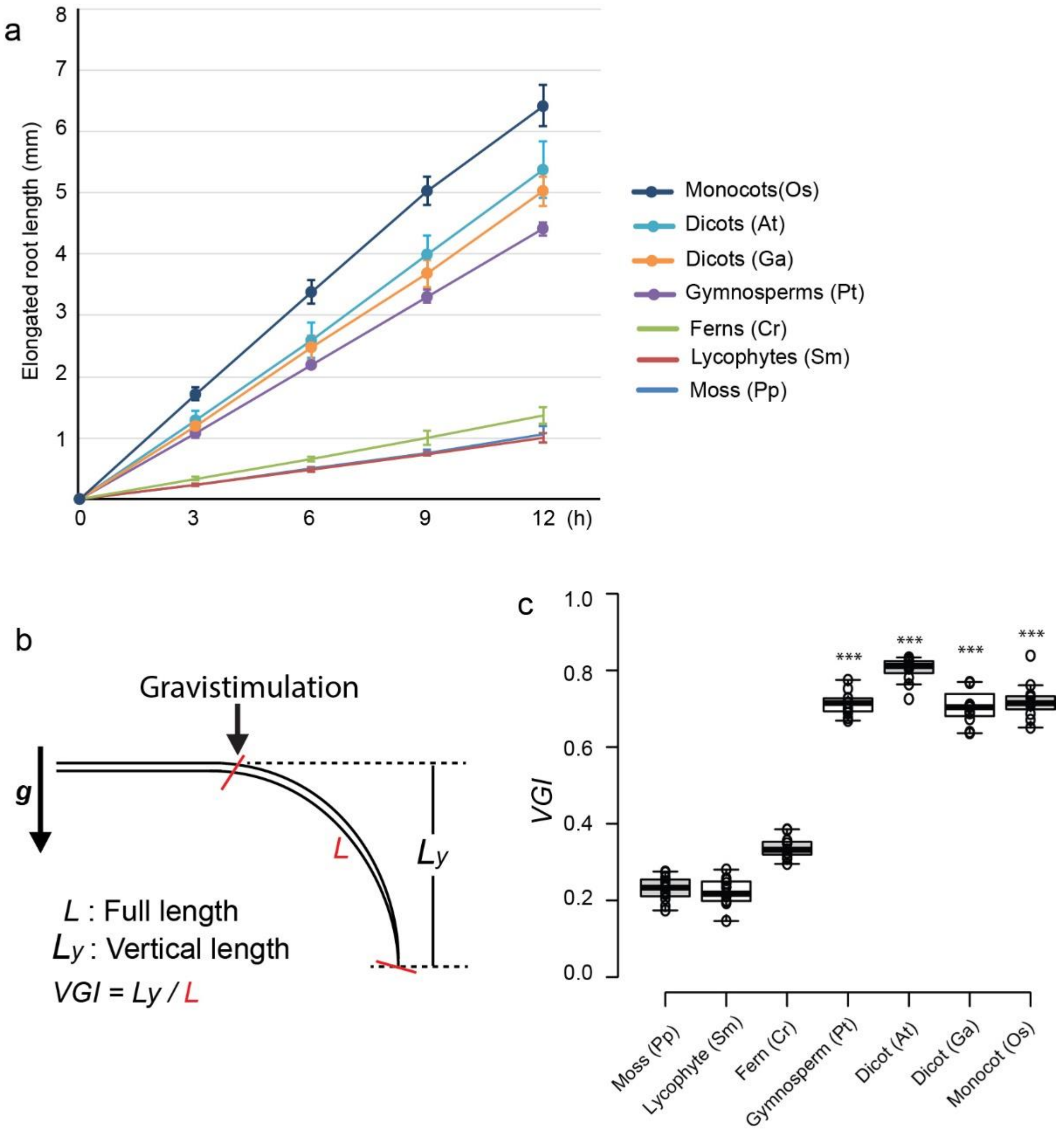

Supplementary Figure 2. The quantification of the rhizoid/roots with the vertical growth index. a, Quantifications of the growth rates for the rhizoid of the moss and the roots of these representative vascular plants (n = 8 roots, graph shows mean length ± SD). **b**, The schematic diagram showing the quantification of gravitropism by analyzing the vertical growth index (VGI) of rhizoid/roots with almost the same elongated length (*L*) after the gravistimulation. **c**, Quantification of the rhizoid/root gravitropism of the diverse plant species with the VGI (as explained in **b**; n ≥ 10 roots). Center lines show the medians; box limits indicate the 25th and 75th percentiles as determined by R software; whiskers extend 1.5 times the interquartile range from the 25th and 75th percentiles, outliers are represented by dots. Student's *t*-test, *** denotes P<0.001,

compared with the basal vascular plant lycophyte (Sm) or ferns (Cr), respectively. Source data are provided as a Source Data file.

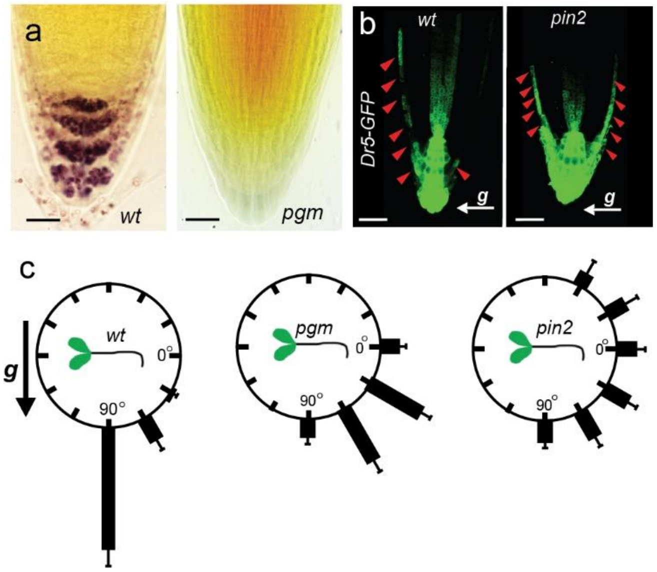

Supplementary Figure 3. The amyloplasts and auxin transporter PIN2 are essential for root gravitropism. a, Starch granules (visualized by Lugol's staining) accumulate within the root apex of wild-type *A. thaliana* (left), while starch granule accumulation is impaired in the *pgm-1* mutant (right). **b**, After gravistimulation for 30 min, activity of the synthetic auxin responsive marker *DR5::GFP* revealed asymmetric auxin distribution in the wild-type *A. thaliana* (left), whereas defects in auxin redistribution were observed in the *pin2* loss-of-function mutant (right). Scale bars, 20 µm in **a** and **b**. **c**, In contrast to the wild type (left), the *pgm-1* (middle) and *pin2* (right) mutants showed impaired root gravitropism after a 90° reorientation of the seedlings for 6 h. Each gravity-stimulated root was assigned to one of 12, 30° sectors. The length of each bar represents the mean percentages ± S.D. of seedlings showing the same direction of root growth of three independent experiments (n ≥ 30 roots).

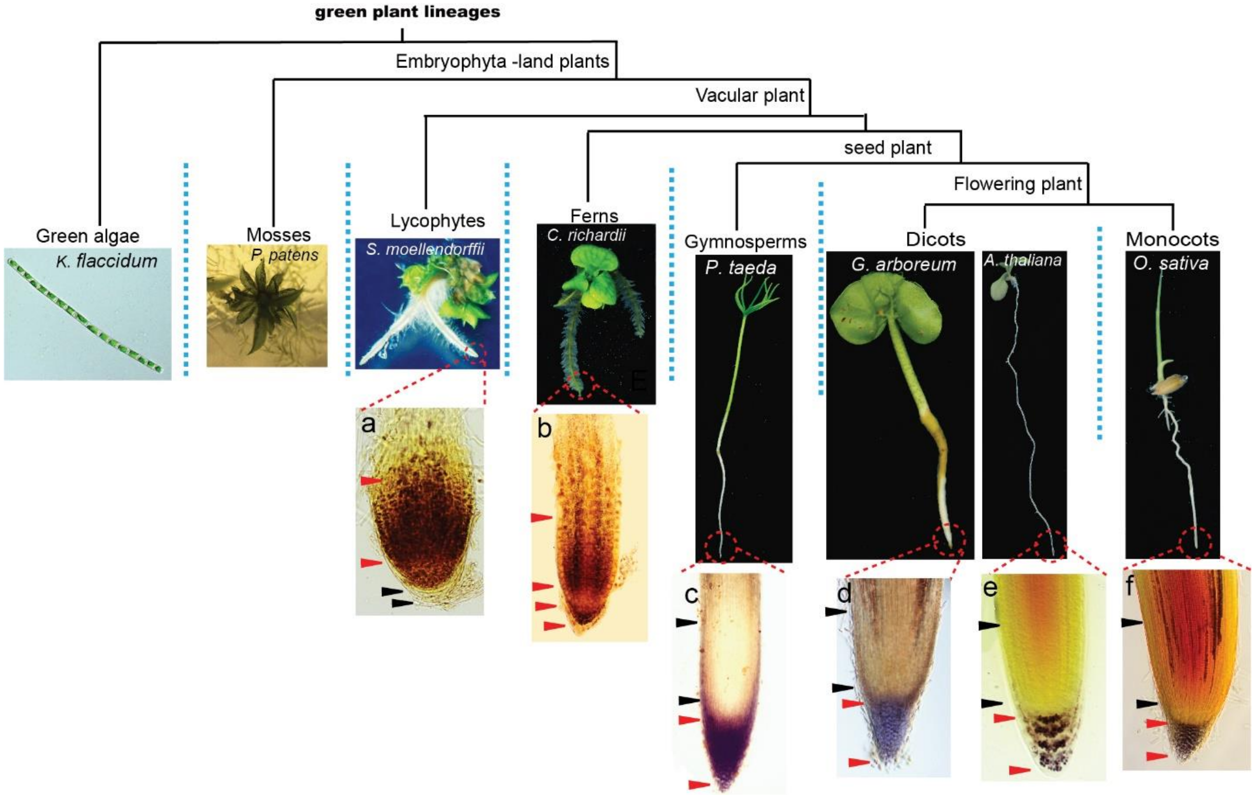

Supplementary Figure 4. Exclusive root apex-specific amyloplast location in the seed plants. a-f, Lugol's staining of the roots from the vascular plants *S. moellendorfﬁi* (lycophyte), *C. richardii* (fern), *P. taeda* (gymnosperm), *G. arboreum* and *A. thaliana* (dicots), and *O. sativa* (monocot). The black arrows indicate the zones lacking starch granules (amyloplasts), and the red arrows indicate the zones containing starch granules.

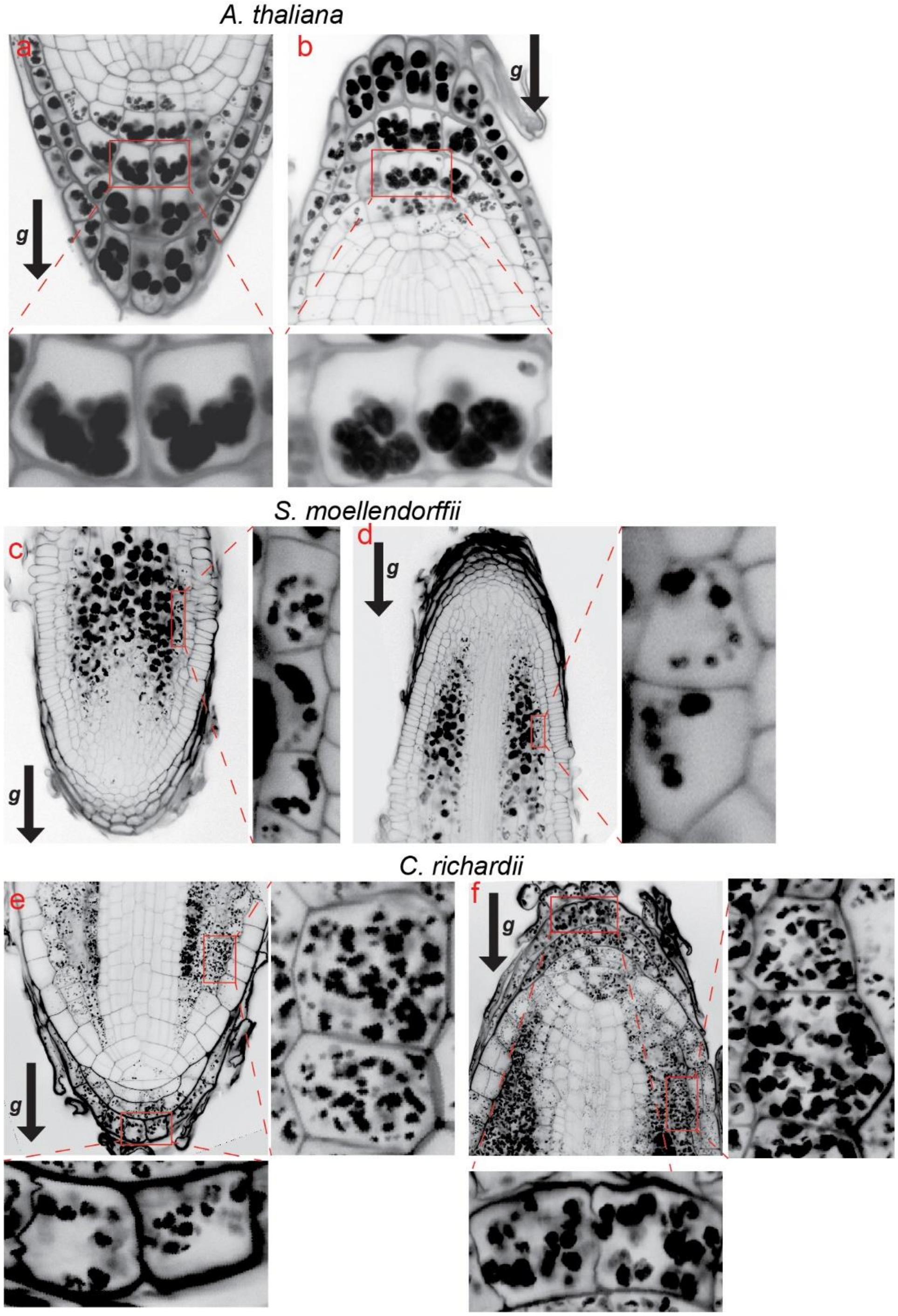

Supplementary Figure 5. Amyloplast sedimentation analysis in the roots of flowering plant *A. thaliana*, lycophyte *S. moellendorffii* and fern *C.richadii*. a-b, mPS-PI staining of the roots from the flowering plant *A. thaliana* showing the amyloplast localization in the root caps before (a) and after a 180° reorientation for 1h (**b**). **c-f**, mPS-PI staining of the roots from the lycophyte *S.*

moellendorffii and fern *C. richardii* roots showing the amyloplast localization before (**c, e**) and after a 180° reorientation of the seedlings for 6h (**d, f**).

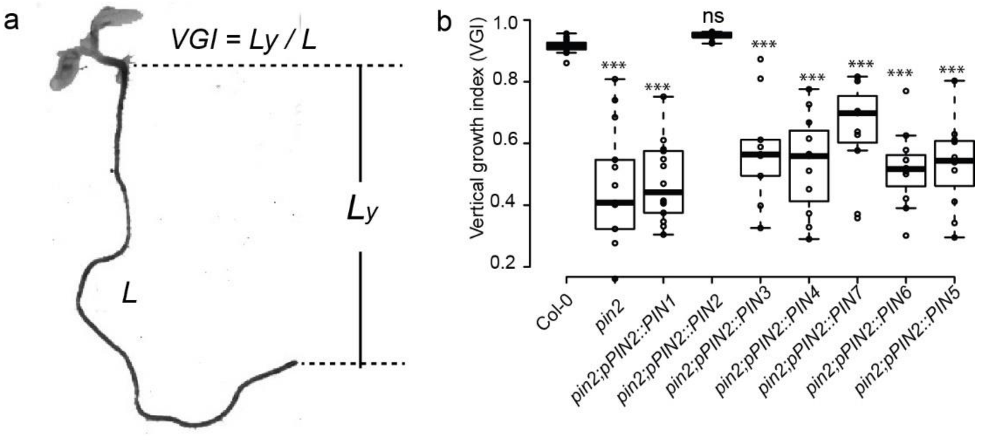

Supplementary Figure 6. The functional specificity of *PIN2* in mediating fast root gravitropism. **a**, **b**, Quantification of the vertical growth index (VGI, as explained in **a**) of the transgenic lines in Fig. 3b-e (n ≥ 10 roots). Center lines show the medians; box limits indicate the 25th and 75th percentiles as determined by R software; whiskers extend 1.5 times the interquartile range from the 25th and 75th percentiles, outliers are represented by dots. Student's *t*-test, *** denotes P<0.001 and ns denotes P>0.05, compared with the *Col-0*, respectively. Source data are provided as a Source Data file.

a

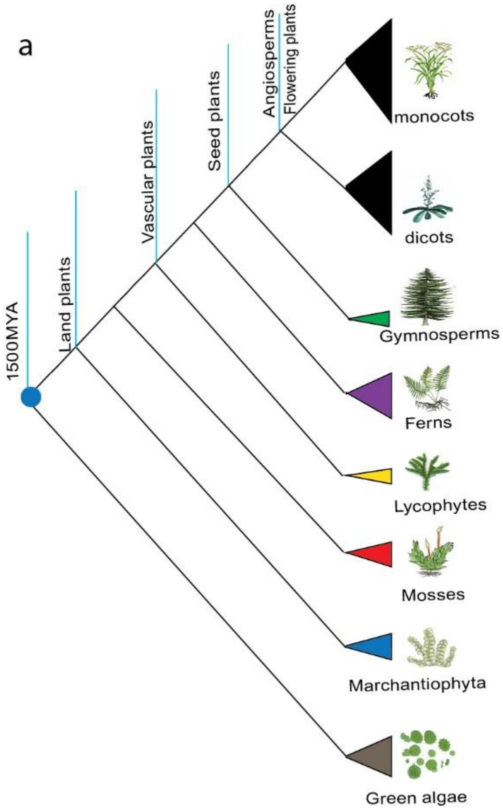

b

	Organism	Number of PIN family proteins	Clades of PINs
Flowering plants	***Monocots***		≥6
	Zea Mays (Zm)	12	
	Oryza sativa (Os)	12	
	Dicots		
	Gossypium arboreum (Ga)	11	
	Arabidopsis thaliana (At)	8	
	Amborella trichopoda (Amt)	9	
	Gymnosperms		4
	Picea abies (Pa)	4	
	Pinus taeda (Pt)	5	
	Ferns		1
	Ceratopteris richardii (Cr)	4	
	Cystopteris fragilis (Cf)	5	
	Lycophytes		1
	Selaginella moellendorffii(Sm)	6	
	Mosses		2
	Physcomitrella patens (Pp)	4	
	Marchantiophyta		
	Marchantia polymorpha (Mp)	4	2
	Green algae		1
	Klebsormidium flaccidum (Kf)	1	
	Mesostigma viride (Mv)	1	

Supplementary Figure 7. Evolution and origin of the *PIN* genes. a, Phylogenetic tree showing the evolutionary relationships between plant lineages. **b,** The number of PIN proteins in various organisms. There are substantially more PIN proteins in the flowering plants than in the other plant lineages (*i.e.,* lycophytes, ferns, gymnosperms, mosses, and green algae). The right column shows the substantially more clades of PIN members in seed plants than other plant lineage according to the phylogenetic tree in the Supplementary Fig. 8.

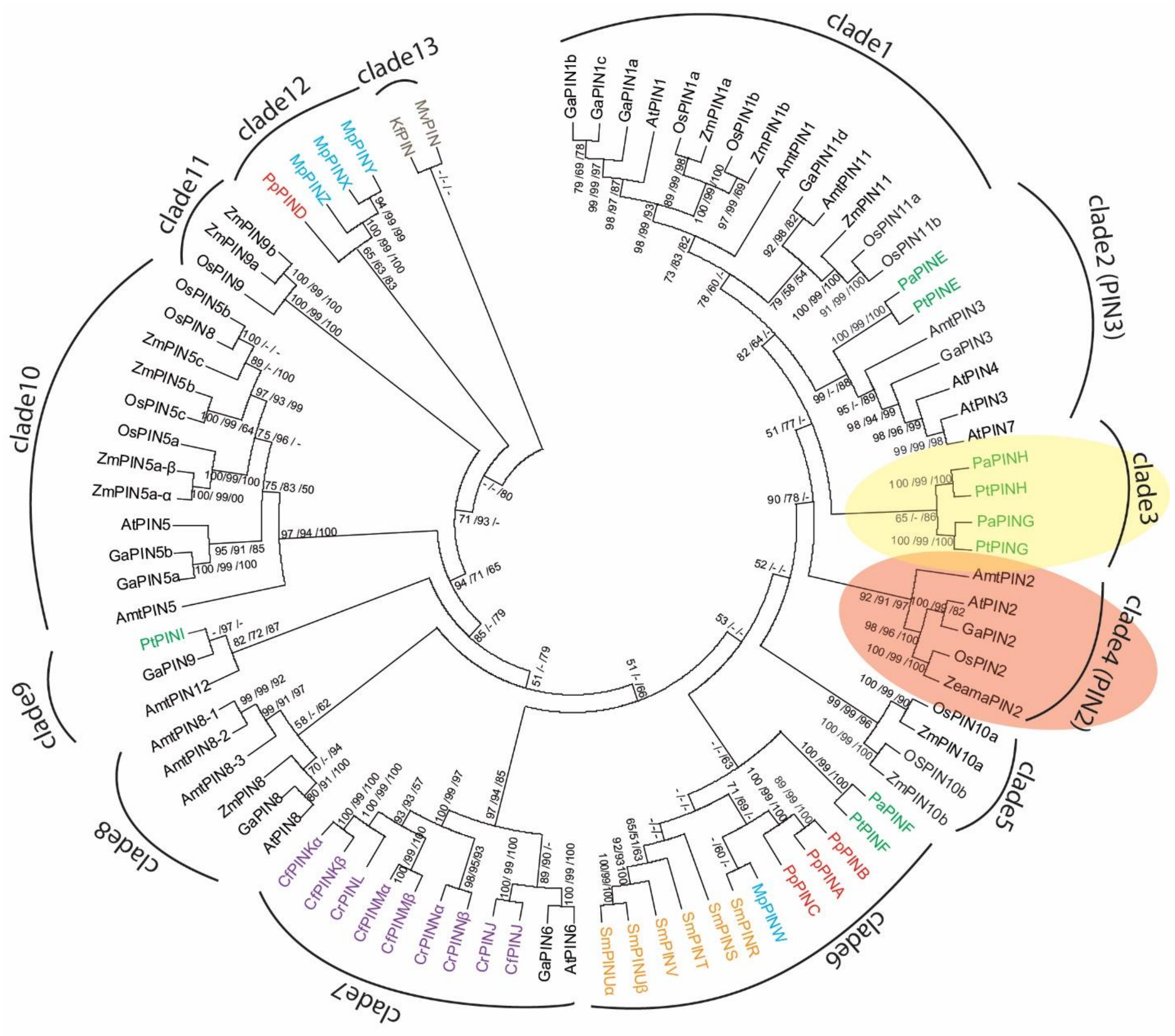

Supplementary Figure 8. Phylogenetic analysis of the plant PIN family. The phylogenetic tree was divided into 13 clades. The red dashed line divides the PINs into two types: canonical PINs and noncanonical PINs. Phylogenetic analysis was performed using the neighbor-joining/maximum-parsimony/maximum-likelihood methods with full-length protein sequences. Bootstrap values are shown on the branch points. Mv, *Mesostigma viride*; Kf, *Klebsormidium flaccidum*; Mp, *Marchantia polymorpha*; Pp, *Physcomitrella patens*; Sm, *Selaginella moellendorffii*; Cr, *Ceratopteris richardii*; Cf, *Cystopteris fragilis*; Pt, *Pinus taeda*; Pa, *Picea abies*; Amt, *Amborella trichopoda*; At, *Arabidopsis thaliana*; Ga, *Gossypium arboreum*; Os, *Oryza sativa*; Zm, *Zea mays*.

The blue colour indicates the marchantiophyte PINs, the red colour indicates the moss PINs, the yellow colour indicates the lycophyte PINs, the purple colour indicates the fern PINs, and the green colour indicates the gymnosperm PINs.

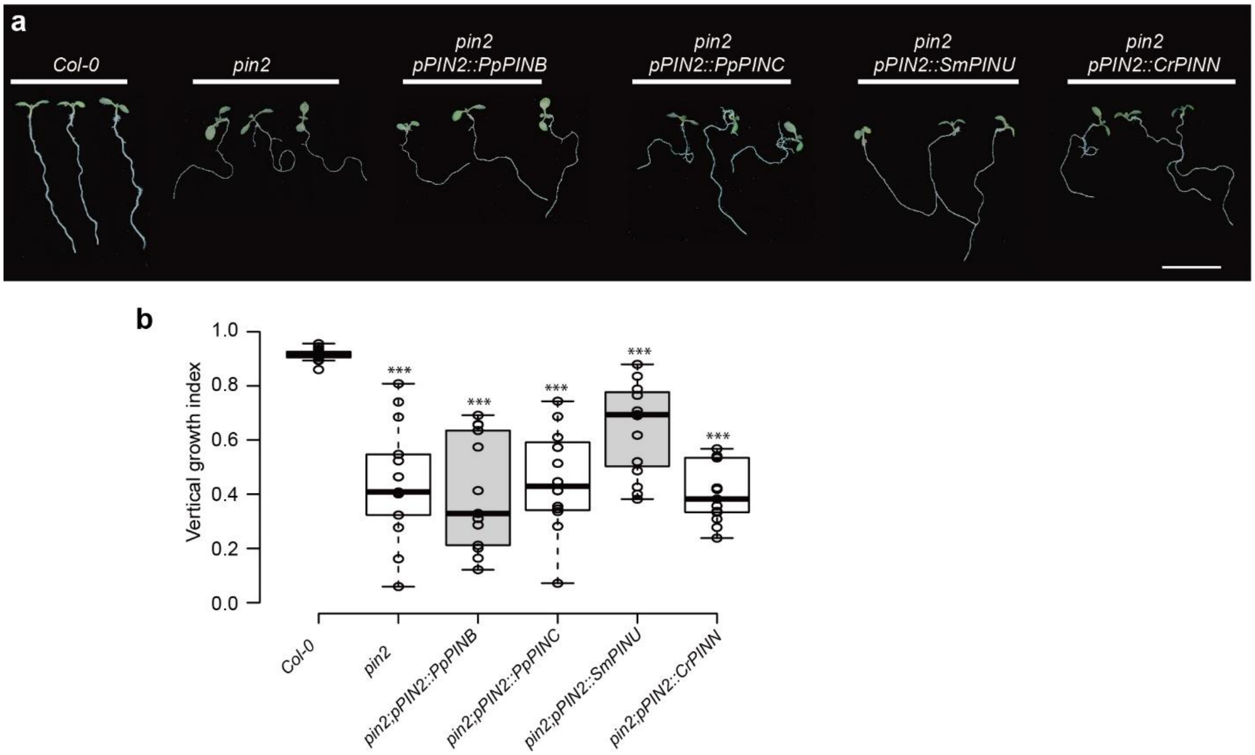

Supplementary Figure 9. PIN2 function in root gravitropism originated in the seed plants. **a**, Interspecies complementation experiments showed that PpPINB, PpPINC (moss), SmPINU (lycophyte) and CrPINN (fern) failed to complement the defective root gravitropism phenotype of the *pin2* mutant. Scale bars, 1 cm. **b**, Quantification of the Vertical growth index (VGI) in **a** ($n \geq 10$ roots). Center lines show the medians; box limits indicate the 25th and 75th percentiles as determined by R software; whiskers extend 1.5 times the interquartile range from the 25th and 75th percentiles, outliers are represented by dots. Student's *t*-test, *** denotes $P<0.001$, compared with the *Col-0*, respectively. Source data are provided as a Source Data file.

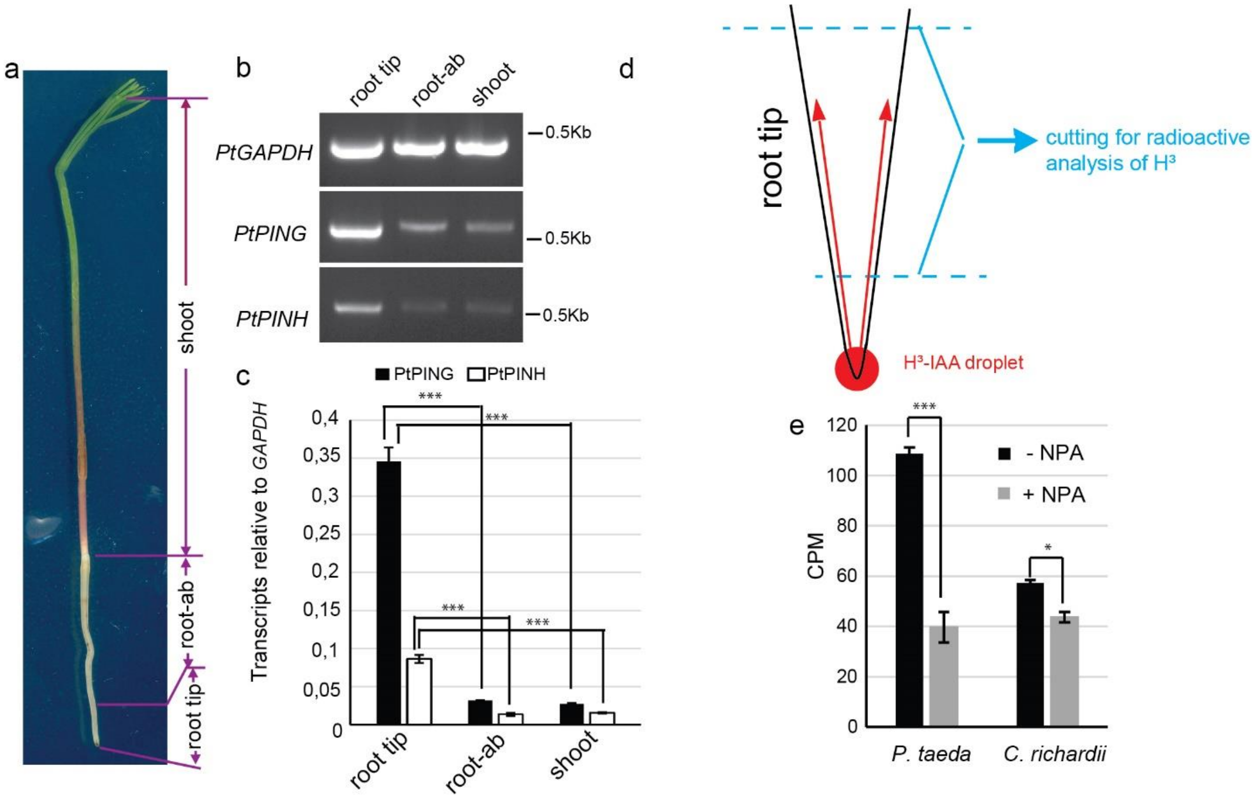

Supplementary Figure 10. The expression of the PING/H and the shootward auxin transport assay in the root tip of *P. taeda*. a-c, Reverse transcription PCR (RT-PCR) (**b**) and quantitative PCR (qPCR) (**c**) analysis of the transcriptional levels of *PtPING/H* in the root tip of *P. taeda* as compared with their transcriptional levels in shoot or in the other part of the root above the root tip (denoted by root-ab) (**a**). Constitutively expressed *PtGAPDH* was used as the internal standard for quantitative analysis. Student's *t*-test, *** denotes P<0.001, compared with the transcription level of *PtPING/PtPINH* in the root tip, respectively. **d,** Schematic diagram showing the measurement of the shootward auxin transport by detecting the ^{3}H labeled IAA (^{3}H-IAA) radioactive intensity of the cut segment of the root (above the root apex) after 6 hour treatment with a 5 μM ^{3}H-IAA droplet in the root apex. **e,** Comparisons of shootward ^{3}H-IAA transport efficiency with or without the treatment of 10μM 1-naphthylphthalamic acid (NPA), an inhibitor of PIN auxin efflux activity, in both the *P. taeda* and *C. richardii* roots. Graph shows mean ± SD from

three independent experiments. Student's *t*-test, *, *** denotes $P<0.01$ or $P<0.001$, compared with the seedlings without the NPA treatment. Source data are provided as a Source Data file.

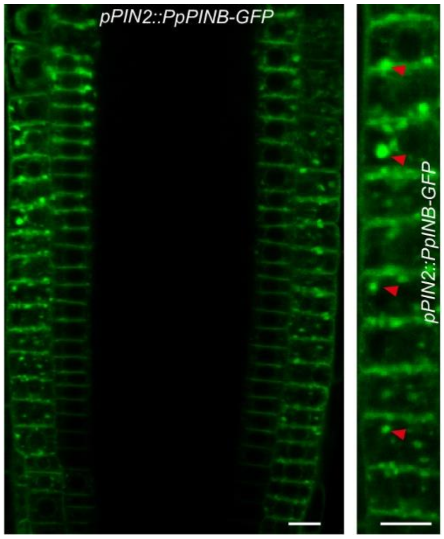

Supplementary Figure 11. The subcellular localization of moss PpPINB expressed under the control of the *Arabidopsis* PIN2 promoter in *Arabidopsis* root epidermal cells. The red arrows indicate aggregated PINB granules in the internal compartments of root epidermal cells. Scale bars, 10 µm.

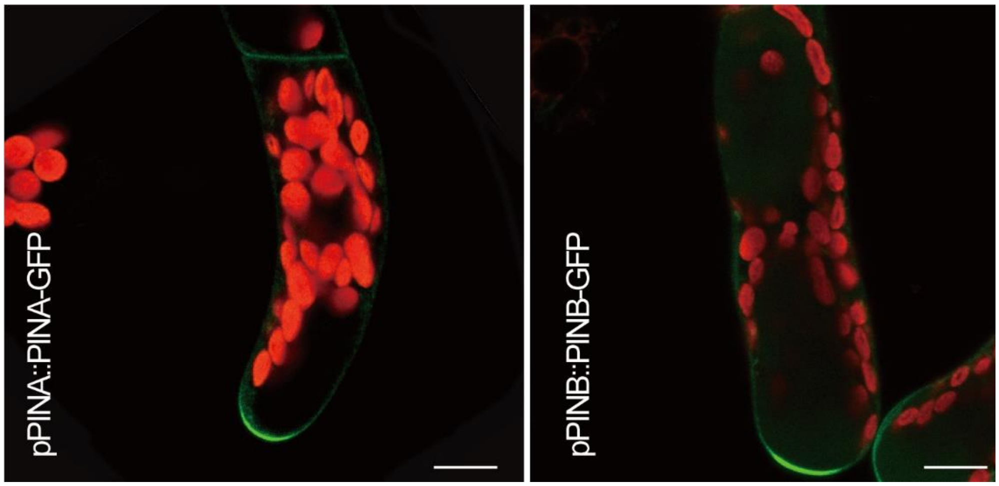

Supplementary Figure 12. The subcellular localization of PpPINA and PpPINB in moss. PpPINA and PpPINB are not localized on the shotward side of rhizoid cells in moss. Scale bars, 10 µm.

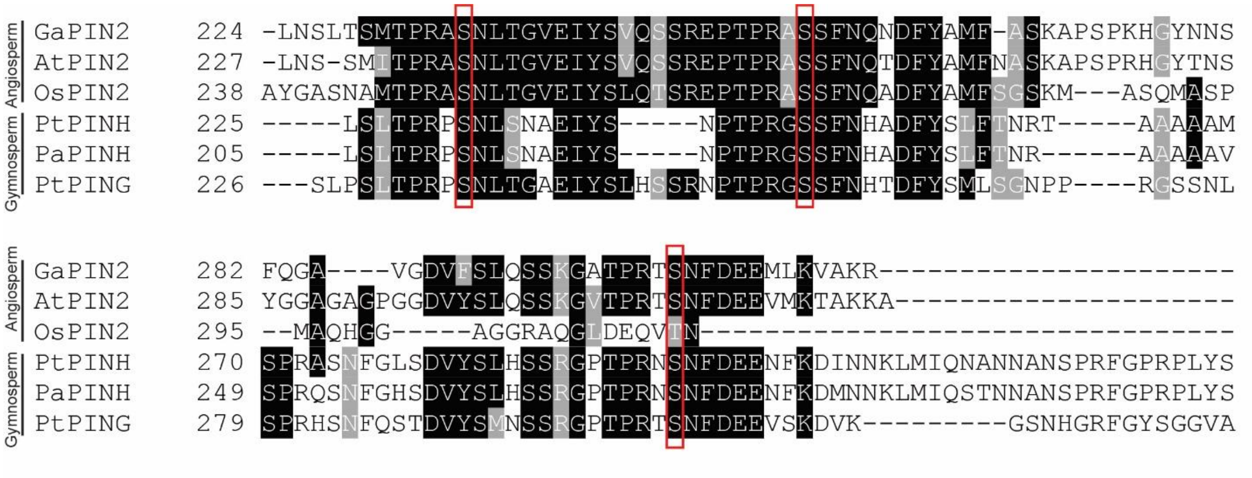

Supplementary Figure 13. The evolutionary conservation of the phosphorylation sites in both the angiosperm PIN2 proteins and gymnosperm PINH/G. The amino acid sequence alignment of the gymnosperm PINH/G and angiosperm PIN2 in the phosphorylation site-spanning region reported by the Dhonukshe *et al*[47], which are critical for the PIN2 function in mediating root gravitropism.

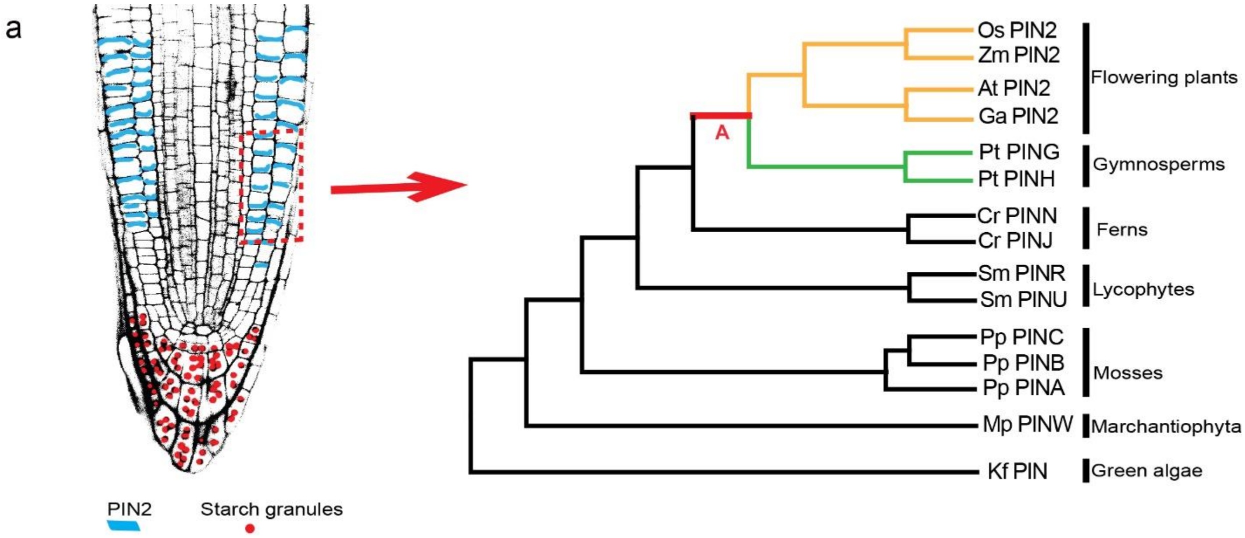

b

Models	Estimates of parameters	Models compared	P values
***PIN* gene dataset**			
A. Branch-site null Model A	ω0=0.07089, ω1=1, ω2=1		
B. Branch-site Model A	ω0=0.06971, ω1=1, ω2=999	B vs. A	0.0016* (<0.01)
C. Three-ratio Model	ω0=0.05769, ω1=999, ω2=0.09591		
D. Four-ratio Model	ω0=0.05771, ω1=999, ω2=0.09095, ω3=0.09730	D vs. C	0.9993 (>0.05)

* Significiant difference between Model B and Model A.

Supplementary Figure 14. Evolution of PIN2 was driven by positive natural selection. a, Phylogeny of *PIN* genes. Left, The schematic drawing of root tip of an *Arabidopsis* seedling. PIN2 expression is indicated by blue lines and starch granule accumulation is indicated by red dots. Right, Phylogenetic tree of PIN proteins from representative plant species. The plant lineages are labeled on the right. **b**, We constrained the branch ancestral to PIN2 and PINH/G (branch A shown in red in Supplementary Fig. 14a) as the foreground and tested branch-site models. In the branch-site null model A (null hypothesis), ω2 for branch A was fixed at 1 (no natural selection), whereas in the branch-site model A (alternative hypothesis), the ω2 for branch A was estimated, and it greatly exceeds 1 (ω2 =999 >1; positive selection). A likelihood ratio test between the two models (alternative hypothesis vs. null hypothesis) was significant (*P* = 0.0016 <0.01) and strongly support the alternative hypothesis, suggesting that positive selection probably increased the evolutionary rate of the *PIN2* and *PINH/G* genes in seed plants

after their separation from the fern lineages. Thus, positive selection may have played an important role in the functional innovations of the *PIN2* and *PINH/G* genes. Then we tested the possibility of differential selection of the *PINH/G* genes in gymnosperms and *PIN2* genes in flowering plants by comparing a three-ratio model with a four-ratio model. In the three-ratio model, three ω values are assumed: ω1 for branch A (red, PIN2 and PINH/G in seed plants) , ω2 was fixed the same for branch in green (PINH/G in gymnosperms) and also for branch in yellow (*PIN2* in flowering plants), and ω0 for all other branches, whereas in the four-ratio model one additional ω, ω3, is assumed for *PIN2* genes (yellow branches), and ω2 is assumed for *PINH/G* genes (green branches) (Supplementary Fig. 14a). Both ω2 (0.09095) and ω3 (0.09730) estimated by the four-ratio model are less than 1, strongly indicative of purifying selection acting on both *PIN2* and *PINH/G* genes. A likelihood ratio test between the two models was not significant (*P* =0.9993 >0.05), suggesting similar levels of purifying selection on the *PIN2* gene in flowering plants and the *PINH/G* genes in gymnosperms.

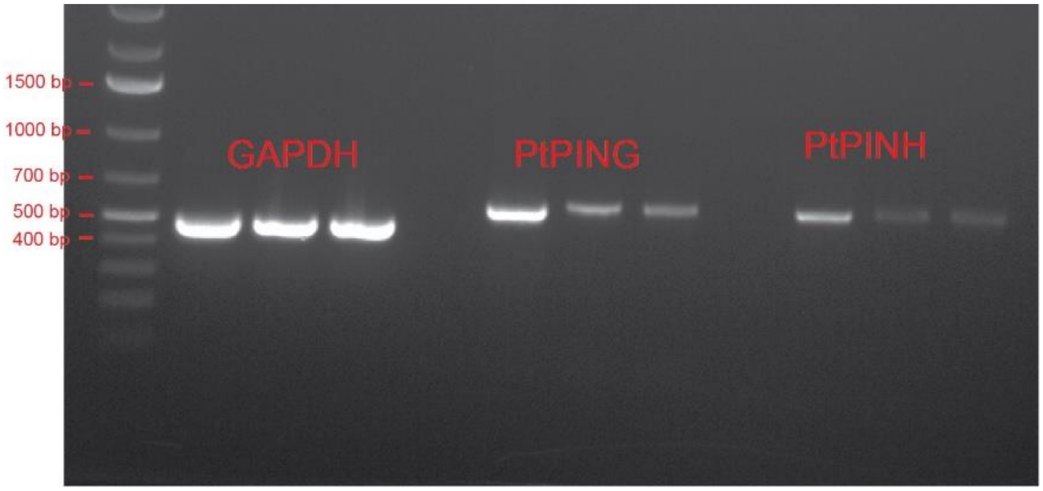

Supplementary Figure 15. Full scan of uncropped gel for Supplementary Fig. 10b.

Supplementary Table 1 | Accession numbers or IDs of *PIN* genes

Protein	Accession no./ID	Protein	Accession no./ID
MpPINZ	Mapoly0089s0050.1	SmPINV	119024
MpPINX	Mapoly0027s0111.1	SmPINUβ	231064
MpPINY	Mapoly0027s0108.1	SmPINUα	268490
MpPINW	Mapoly0053s0108.1	SmPINS	234325
CfPINMα	scaffold-LHLE-2007361	ZmPIN2	AQL05162.1
CfPINMβ	scaffold-LHLE-2016421	ZmPIN1a	GRMZM2G149184_T01
CfPINKα	scaffold-LHLE-2003173	ZmPIN1b-α	GRMZM2G149184_T01
CfPINKβ	scaffold-LHLE-2003174	ZmPIN1b-β	GRMZM2G098643_T01
CfPINJ	scaffold-LHLE-2011490	ZmPIN10a	GRMZM2G126260_T02
PaPINE	lcl|MA_100472g0010	ZmPIN10b	GRMZM2G160496_T01
PaPINH	lcl|MA_69724g0010	ZmPIN11	GRMZM2G171702_T01
PaPING	lcl|MA_61553g0010	ZmPIN5a-α	GRMZM2G025742_T01
PaPINF	lcl|MA_10258002g0010	ZmPIN5a-β	GRMZM2G175983_T01
PtPINE	lcl|PITA_000003882	ZmPIN5b	GRMZM2G040911_T01
PtPINH	lcl|PITA_000024726	ZmPIN5c	GRMZM2G148648_T01
PtPING	lcl|PITA_000013100	ZmPIN8	GRMZM5G839411_T02
PtPINF	lcl|PITA_000046447	ZmPIN9	GRMZM5G859099_T01
PtPINI	lcl|PITA_000012975	OsPIN1b	XP_015627099.1
AmtPIN1	lcl|evm_27.model.AmTr_v1.0_scaffold00010.98	OsPIN1a	XP_015641301.1
AmtPIN3	lcl|evm_27.model.AmTr_v1.0_scaffold00024.330	OsPIN10a	XP_015625677.1
AmtPIN11	lcl|evm_27.model.AmTr_v1.0_scaffold00101.123	OsPIN11a	XP_015616014.1
AmtPIN2	lcl|evm_27.model.AmTr_v1.0_scaffold00019.408	OsPIN11b	XP_015619425.1
AmtPIN12	lcl|evm_27.model.AmTr_v1.0_scaffold00013.203	OsPIN2	XP_015641653.1
AmtPIN8-1	lcl|evm_27.model.AmTr_v1.0_scaffold00038.32	OsPIN10b	XP_015638890.1
AmtPIN8-2	lcl|evm_27.model.AmTr_v1.0_scaffold00038.35	OsPIN8	XP_015649668.1
AmtPIN5	lcl|evm_27.model.AmTr_v1.0_scaffold00013.199	OsPIN5b	XP_015649102.1
AmtPIN8-3	lcl|evm_27.model.AmTr_v1.0_scaffold00038.38	OsPIN5c	XP_015611661.1

SmPINR	99301	OsPIN5a	XP_015621857.1
SmPINT	102666	OsPIN9	XP_015634219.1

Mp: *Marchantia polymorpha* Cf: *Cystopteris fragilis*

Pa: *Picea abies* Pt: *Pinus taeda*

Sm: *Selaginella moellendorffii* Amt: *Amborella trichopoda*

Zm: *Zea Mays* Os: *Oryza sativa*
