# Supplementary material for: Evolution of fast root gravitropism in seed plants
Source: Nat Commun. 2019 Aug 2;10:3480. doi: 10.1038/s41467-019-11471-8 (PMC6677796; doi:10.1038/s41467-019-11471-8)
Supplement: Supplementary file 3 — Description of Additional Supplementary Files [file 41467_2019_11471_MOESM3_ESM.pdf]

Description of Additional Supplementary Files

File Name: Supplementary Data 1
Description: Primers used for vector construction
