# Supplementary material for: Evolution of fast root gravitropism in seed plants
Source: Nat Commun. 2019 Aug 2;10:3480. doi: 10.1038/s41467-019-11471-8 (PMC6677796; doi:10.1038/s41467-019-11471-8)
Supplement: Supplementary file 4 — Supplementary Data 1 [file 41467_2019_11471_MOESM4_ESM.docx]

Primers used for vector construction

| Gene | Primer sequences |
| --- | --- |
| Primer sequences used for the interspecies genetic complementation | |
| *PIN2* promoter | 5′- TATAGAAAAGTTGTAAATAGTTTCATCCTGTTTTATCAGGCTACATTCAC -3′  5′- TTTGTACAAACTTGATTTGATTTACTTTTTCCGGCGAGAGAGAGAAGAAG -3′ |
| *A. thanlian PIN1* CDS | 5′- AAAAAAGCAGGCTTCATGATTACGGCGGCGGACTTCTACCA -3′  5′- CAAGAAAGCTGGGTTTCATAGACCCAAGAGAATGTAGTAGA -3′ |
| *A. thanlian PIN2* CDS | 5′- AAAAAAGCAGGCTTCATGATCACCGGCAAAGACATGTACGA -3′  5′- CAAGAAAGCTGGGTTTTAAAGCCCCAAAAGAACGTAGTAGAG -3′ |
| *A.thanlian PIN3* CDS | 5′- AAAAAAGCAGGCTTCATGATCTCATGGCACGACCTCTACAC -3′  5′- CAAGAAAGCTGGGTTTTATAACCCGAGTAGAATGTAGTAAA -3′ |
| *A.thanlian PIN4* CDS | 5′- AAAAAAGCAGGCTTCATGATTACGTGGCACGACTTGTACAC -3′  5′- CAAGAAAGCTGGGTTTCAAAGGCCAAGAAGAATATAGTAGA -3′ |
| *A.thanlian PIN5* CDS | 5′- AAAAAAGCAGGCTTCATGATAAATTGTGGAGATGTTTACAA -3′  5′- CAAGAAAGCTGGGTTTCAATGAATAAACTCCAGAGCTGCGT -3′ |
| *A.thanlian PIN6* CDS | 5′- AAAAAAGCAGGCTTCATGATAACGGGAAACGAATTCTACAC -3′  5′- CAAGAAAGCTGGGTTTCATAGGCCCAAGAGGACGTAGTACA -3′ |
| *A.thanlian PIN7* CDS | 5′- AAAAAAGCAGGCTTCATGATCACATGGCACGACCTCTACAC -3′  5′- CAAGAAAGCTGGGTTTTATAGCCCGAGTAAAATGTAGTAAA -3′ |
| *KfPIN* CDS | 5′-: AAAAAAGCAGGCTTCATGGCATCCGGCGGCCATGGCAGCAT-3′  5′- CAAGAAAGCTGGGTTTCAGAAGTGTTCCAGCGCGACGTAGT-3′ |
| *PpPINA* CDS | 5′- AAAAAAGCAGGCTTCATGATTAACGGCCATGACATATACAA -3′  5′- CAAGAAAGCTGGGTTTCACAGACCAAGTAATATGTAGTAT -3′ |
| *PpPINB* CDS | 5′- AAAAAAGCAGGCTTCATGATTAACGGGCATGACATTTACAA -3′  5′- CAAGAAAGCTGGGTTTCACAGACCAAGTAATATGTAGTACA -3′ |
| *MpPINZ* CDS | 5′- AAAAAAGCAGGCTTCATGATCAACGCGCACGATCTCTACAA-3′  5′- CAAGAAAGCTGGGTTTTACAGGCCCAAAAGTACGTAGTACA-3′ |
| *CrPINN* CDS | 5′- AAAAAAGCAGGCTTCATGATAACAATAAACGACTTTTAT-3′  5′- CAAGAAAGCTGGGTTTTATATTCCCAGAAAGACATAGTA -3′ |
| *CrPINJ* CDS | 5′- AAAAAAGCAGGCTTCATGATTACGGGCAAGGACATGTATGAT-3′  5′- CAAGAAAGCTGGGTTTCAGATTCCCAGAAGCACGTAGTA-3′ |
| *SmPINR* CDS | 5′- AAAAAAGCAGGCTTCATGATAAGTCTCCATGATCTCTACAC -3′  5′- CAAGAAAGCTGGGTTCTACAGACCCAAGAGCACGTAATACA -3′ |
| *SmPINU* CDS | 5′- AAAAAAGCAGGCTTCATGATAAGTCCGGCGGAGTTCTACTC-3′  5′- CAAGAAAGCTGGGTTCTATAATCCCAGAAGCACGTAATAGA -3′ |
| *PtPINH* CDS | 5′- AAAAAAGCAGGCTTCATGATTAATGGAGCGGACATATAC-3′  5′- CAAGAAAGCTGGGTTTCAAACTCCAAGAAGAATGTAATA-3′ |
| *PtPINE* CDS | 5′- AAAAAAGCAGGCTTCATGATAAAGGCCGGGGATCTGTAT-3′  5′- CAAGAAAGCTGGGTTTCAGAGGCCCAACACAACGTAGTA -3′ |
| *PtPING* CDS | 5′- AAAAAAGCAGGCTTCATGATTACCTTGAAGGATTTGTAT-3′  5′- CAAGAAAGCTGGGTTTCATGATAATGAATTTAGATCCCT-3′ |
| *PtPINI* CDS | 5′- AAAAAAGCAGGCTTCATGATAACTTGGAAGGATTTATAC-3′  5′- CAAGAAAGCTGGGTTTCACGAGTGGACTTCAAGAAGGAC-3′ |
| Primer sequences used for the PIN-GFP fusion with overlapping PCR | |
| *PtPINH-Fragment1* | 5′- AAAAAAGCAGGCTTCATGATTAATGGAGCGGACAT -3′  5′- TGAACAGCTCCTCGCCCTTGCTCACCATATTACTGTTATGGTTGTTGT -3′ |
| *GFP* | 5′- ATGGTGAGCAAGGGCGAGGAGCT-3′  5′- CTTGTACAGCTCGTCCATGC-3′ |
| *PtPINH-Fragment3* | 5′- GCATGGACGAGCTGTACAAGTCCCATGTCAAGGATATACG -3′  5′- CAAGAAAGCTGGGTTTCAAACTCCAAGAAGAATGT -3′ |
| *PtPING-Fragment1* | 5′- AAAAAAGCAGGCTTCATGATTACCTTGAAGGATTT -3′  5′- CCTCGCCCTTGCTCACCATATTGTTATCAGGTGGGGCTC -3′ |
| *PtPING-Fragment3* | 5′- GCATGGACGAGCTGTACAAGGGTAAAAGTGATCCAAATGG -3′  5′- CAAGAAAGCTGGGTTTCATGATAATGAATTTAGAT -3′ |
| *PtPINE-Fragment1* | 5′- AAAAAAGCAGGCTTCATGATAAAGGCCGGGGATCT -3′  5′- CCTCGCCCTTGCTCACCATCCGACTCGATATCACAGACC -3′ |
| *PtPINE-Fragment3* | 5′- GCATGGACGAGCTGTACAAGAGATCCATGGGGTTGGGCTC -3′  5′- CAAGAAAGCTGGGTTTCAGAGGCCCAACACAACGT -3′ |
| *PtPINI-Fragment1* | 5′- AAAAAAGCAGGCTTCATGATAACTTGGAAGGATTT -3′  5′- CCTCGCCCTTGCTCACCATCACTGCAGCTGCATGTGGAG -3′ |
| *PtPINI-Fragment3* | 5′- GCATGGACGAGCTGTACAAGATGAGAATAATATTAGGCAT -3′  5′- CAAGAAAGCTGGGTTTCACGAGTGGACTTCAAGAA -3′ |
| *CrPINJ-Fragment1* | 5′- AAAAAAGCAGGCTTCATGATTACGGGCAAGGACAT -3′  5′- CCTCGCCCTTGCTCACCATCATGATAGTTGATGAAGGAA -3′ |
| *CrPINJ-Fragment3* | 5′- GCATGGACGAGCTGTACAAGATAAAGCTCATACTCCAAAT -3′  5′- CAAGAAAGCTGGGTTTCAGATTCCCAGAAGCACGT -3′ |
| *SmPINR-Fragment1* | 5′- AAAAAAGCAGGCTTCATGATAAGTCTCCATGATCT -3′  5′- CCTCGCCCTTGCTCACCATCATAATCCGAGCCGGAGGCA -3′ |
| *SmPINR-Fragment3* | 5′- GCATGGACGAGCTGTACAAGACGAGGGTGATACTGAGCAT -3′  5′- CAAGAAAGCTGGGTTCTACAGACCCAAGAGCACGT -3′ |
| *MpPINZ-Fragment1* | 5′- AAAAAAGCAGGCTTCATGATCAACGCGCACGATCT -3′  5′- CCTCGCCCTTGCTCACCATCACTCTCGAAGGAGGCATGC -3′ |
| *MpPINZ-Fragment3* | 5′- GCATGGACGAGCTGTACAAGATGATCCGGCTGATTTTGGA -3′  5′- CAAGAAAGCTGGGTTTTACAGGCCCAAAAGTACGT -3′ |
| *Primer sequences used for the swapping of PIN2 HL with other PIN* | |
| *KfPIN-N-TMD* | 5′- AAAAAAGCAGGCTTCATGGCATCCGGCGGCCATG -3′  5′- CTGCTCGGAGATGAGAAGTCGGGACGCCCGAATCTC -3′ |
| *AtPIN2-HL* | 5′- CTTCTCATCTCCGAGCAG -3′  5′- CATTATCAGAATTAGTCT -3′ |
| *KfPIN-C-TMD* | 5′- AGACTAATTCTGATAATG GGGTGGAAGCTGCGCAAG -3′  5′- CAAGAAAGCTGGGTTTCAGAAGTGTTCCAGCGC -3′ |
| *MpPINZ-N-TMD* | 5′- AAAAAAGCAGGCTTCATGATCAACGCGCACGATCTC -3′  5′- CTGCTCGGAGATGAGAAGCTTGGCCGCGCGGAACTC -3′ |
| *MpPINZ-C-TMD* | 5′- AGACTAATTCTGATAATGGTTTTCCGCAAGCTCGTC -3′  5′- CAAGAAAGCTGGGTTTTACAGGCCCAAAAGTAC -3′ |
| *SmPINR-N-TMD* | 5′- AAAAAAGCAGGCTTCATGATAAGTCTCCATGAT -3′  5′- CTGCTCGGAGATGAGAAGCTTCGCGCTGCGGTACTC -3′ |
| *SmPINR-C-TMD* | 5′- AGACTAATTCTGATAATGGTGTGGCGAAAGCTTGTT -3′  5′- CAAGAAAGCTGGGTTCTACAGACCCAAGAGCAC -3′ |
| *CrPINJ- N-TMD* | 5′- AAAAAAGCAGGCTTCATGATTACGGGCAAGGAC -3′  5′- CTGCTCGGAGATGAGAAGCCTCGCCGCCCGTAGCTC -3′ |
| *CrPINJ-C-TMD* | 5′- AGACTAATTCTGATAATGGTACTACGTAAGCTGATT -3′  5′- CAAGAAAGCTGGGTTTCAGATTCCCAGAAGCAC -3′ |
| *PtPING-HL* | 5′- ATGCTTATCATGGAGCAGTT -3′  5′- CATTATGAGAATTAGCCTAG -3′ |
